# Compositional schedulability analysis of real-time actor-based systems

**DOI:** 10.1007/s00236-015-0254-x

**Published:** 2016-01-25

**Authors:** Mohammad Mahdi Jaghoori, Frank de Boer, Delphine Longuet, Tom Chothia, Marjan Sirjani

**Affiliations:** 1 AMC, Amsterdam, The Netherlands; 2 CWI, Amsterdam, The Netherlands; 3 Leiden University, Leiden, The Netherlands; 4 University Paris-Sud, LRI UMR8623, 91405 Orsay, Paris, France; 5 School of Computer Science, University of Birmingham, Birmingham, UK; 6 Reykjavík University, Reykjavík, Iceland; 7 University of Tehran and IPM, Tehran, Iran

## Abstract

We present an extension of the actor model with real-time, including deadlines associated with messages, and explicit application-level scheduling policies, e.g.,“earliest deadline first” which can be associated with individual actors. Schedulability analysis in this setting amounts to checking whether, given a scheduling policy for each actor, every task is processed within its designated deadline. To check schedulability, we introduce a compositional automata-theoretic approach, based on maximal use of model checking combined with testing. Behavioral interfaces define what an actor expects from the environment, and the deadlines for messages given these assumptions. We use model checking to verify that actors match their behavioral interfaces. We extend timed automata refinement with the notion of deadlines and use it to define compatibility of actor environments with the behavioral interfaces. Model checking of compatibility is computationally hard, so we propose a special testing process. We show that the analyses are decidable and automate the process using the Uppaal model checker.

## Introduction

Actors were originally introduced by Hewitt as autonomous reasoning objects [33]. Actor languages have since then evolved as a powerful tool for modeling distributed and concurrent systems [3, 4]. Different extensions of Actors are proposed in several domains and are claimed to be the most suitable model of computation for many dominating applications [34]. Examples of these domains include designing embedded systems [50, 51], wireless sensor networks [19], multi-core programming [43] and designing web services [16, 17].

In an Actor-based model, actors are (re)active objects with encapsulated data and methods which represent their state and behavior, respectively. Actors are the units of concurrency, i.e., an actor conceptually has a dedicated processor. In the pure asynchronous setting [3, 33], actors can only send asynchronous messages and have queues for receiving messages. An actor progresses by taking a message out of its queue and processing it by executing its corresponding method. A method is a piece of sequential code that may send messages. We model dynamic reconfiguration by deciding the message recipients based on actor states, but we do not consider dynamic creation of actors. We may use the terms actors and objects interchangeably.

This model of concurrent computation forms the basis of the programming languages Erlang [9] and Scala [30] that have recently gained in popularity, in part due to their support for scalable concurrency. However, for optimal use of both hardware and software resources, we cannot avoid leveraging scheduling and performance related issues from the underlying operating system to the application level as argued for example in [8, 11]. In general, the goal of *resource-aware programming* (RAP [54], SUMATRA [2], CAMELOT [52]) is to express policies for the management of resources.

In this paper, we focus on CPU time and introduce a new extension of the actor model with real-time and present a methodology for compositional schedulability analysis. The key to compositionality is the actors’ behavioral interfaces where the communication protocol of each actor is defined in terms of how it is expected to send and receive messages. In the first step for compositional analysis, as previously introduced in [38], every actor is individually analyzed for schedulability by taking its behavioral interface as an abstraction of the environment. However, at the next step of composing the actors, we need to make sure that each actor’s environment is indeed compatible with the protocol specified in its behavioral interface. As discussed in Sect. 4, it does not suffice to check compatibility only at the level of behavioral interfaces and we did not address this problem in our previous work [38] due to its complexity. This paper, as the extended version of our initial report in [40], proves that the notion of refinement including quiescence and deadlines is a sufficient condition for compositionality, even in presence of dynamic reconfiguration. Nevertheless since this approach still requires reasoning on the global system, we propose a testing method. By using Uppaal model checker for testing, we aims at finding counter-examples to refinement, which may in turn be used as test cases for global schedulability. In the rest of this section, we present an intuitive introduction to our methodology for modeling and the schedulability analysis framework and finally, at the end of this section, the extension points with respect to our initial report in [40] are identified.

**Fig. 1:**
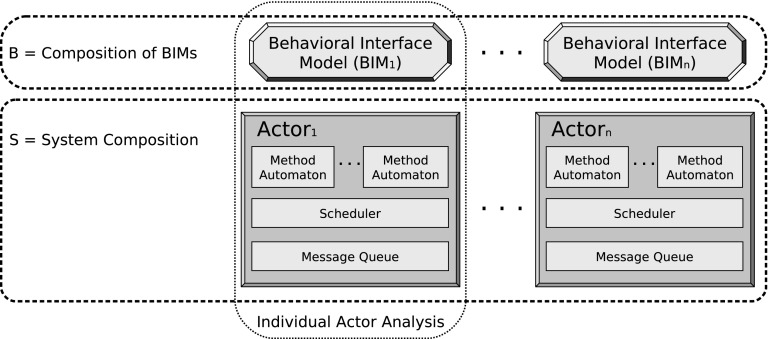
An off-the-shelf actor component is guaranteed to be schedulable if it is used as expected in its behavioral interface. This correct usage in a system *S* can be tested, called the compatibility check

Figure 1 presents the two levels of abstraction we propose for modeling real-time actors. At the detailed level, an actor consists of its methods, scheduler and queue. This specification is given in timed automata [7] in order to support automated analysis techniques. In this model, deadlines are assigned to messages and explicit application-level scheduling policies are associated to the individual actors (rather than, for instance, assuming “First Come First Served” (FCFS) by default). A scheduling policy determines the order in which the (methods corresponding to) queued messages should be executed. We restrict to schedulers in which a new message cannot preempt the currently running method; as we discussed in [37, 38], preemptive scheduling is undecidable in this framework unless a minimum delay is enforced between every two rounds of preemption. Method automata mainly specify what messages are sent and when. Received messages are handled by the schedulers and can be seen as events generating tasks (as a comparison to Task Automata [25]). A deadline is assigned to each message specifying the time before which the intended job should be accomplished. Although we ignore communication delays, they can be added as we considered in [39]. This framework allows quality-of-service and deployment requirements to be analyzed and resolved at design time. For example in a real-time setting, we must guarantee a maximum on average response-time (end-to-end deadlines) or a minimum on the level of system throughput. For our analysis, we have chosen to use Uppaal  [49]; this choice is however not essential and in principle other tools for timed automata can also be employed. This framework complements the work in [57], in which we describe how application-level scheduling policies can be implemented into a programming language on top of Java. To exemplify our approach, we model in Sect. 3.2 a peer-to-peer system with an architecture similar to that of Skype: a centralized broker is responsible for connecting the clients (aka peers). The goal is to ensure that the system will function quickly without delays. The analysis is difficult because this is a large distributed system; furthermore, the network of the connected peers may change dynamically.

As seen in Fig. 1, a behavioral interface provides an abstract and high-level view of the actor by abstracting from the queue and method implementations. In fact, it shows the pattern of possible interactions it may have with its environment. This captures the valid sequences of provided and required services (received and sent messages). We model a behavioral interface as a deterministic timed automaton to further capture the timings and deadlines of the messages. For instance, the behavioral interface of a server that handles at most one request at a time can be defined as a loop of receiving a ‘request’ followed by a ‘reply’, i.e., no second request is allowed before providing a reply.

To perform schedulability analysis of an actor-based system in a compositional manner, first schedulablity of every actor has to be analyzed individually with respect to its behavioral interface. This was elaborated in our previous work [38], which is based on the ideas of Task Automata [25]. As an actor is expected to be used as specified by its behavioral interface, we restrict the actor behavior accordingly when checking its schedulability in isolation. Our method allows us to statically find an upper bound on the length of schedulable queues; hence, the behavioral model of the actor has a finite number of states and the analysis is decidable. This way, one can analyze the actor with regard to different scheduling strategies, and find the best strategy.

Once an actor is proved schedulable, in order to use it as an off-the-shelf component we need to check additionally that its actual usage in a given distributed system follows the expected usage (as specified by the behavioral interface), called the compatibility check. In other words, checking compatibility for each actor $A_i$ involves ensuring that the rest of the system respects the requirements specified in its behavioral interface $B_i$. Unfortunately, as we explain in Sect. 4, this cannot be checked simply at the level of behavioral interfaces, because they include only an approximation of when messages are sent.

In theory, compatibility can be checked by to constructing the complete system behavior *S* of all actors together and showing that *S* is a *refinement* of the product of the automata for behavioral interfaces (call it *B*), i.e., the set of timed traces of *S* is a subset of that of *B*. Assuming that *B* is deterministic, one can prove that *S* is refinement of *B* by model-checking the synchronous product of *S* and *B* (restricted by the computed queue bounds). Due to the message queues (one for each actor) in *S*, this would lead however to an unmanageable state-space explosion. In [36], we have investigated a compositional approach purely based on model checking for schedulability and compatibility analysis, but to preserve soundness, it becomes pessimistic. Instead of verifying the refinement relation, we introduce a novel method for *counter-example oriented* testing, which is more realistic. In this method we generate a test case from *B* as follows. We take a trace from *B* and complete it into a test case for *S* by adding transitions that capture all possible one-step deviations from the original trace. Among these transitions, those not allowed in *B* produce a counter-example, i.e., *a trace of S which does not belong to B*. This technique is much more effective than generating test cases from *S* to be checked against *B*, because it allows for automated generation of test cases from *B* (note that *B* does not involve queues) and a reduction of the overall system behavior *S* by the test case.

Our proposed testing technique gives rise to some issues which are not common in standard frameworks. First, the system under test is a model and not a real implementation. We do not take advantage of our knowledge of the model, so it can be seen as black-box testing, but the execution of test cases will be simplified as we can apply tools that can systematically explore a model. Another difference from usual frameworks is that the system involves internal actions that are not specified in the behavioral interfaces. The consequence is that the test, built from the abstract specification only, will not be able to fully control the system under test during its execution. This leads to a lot of non-determinism but this can be solved by using a model checker to execute a test case. Last but not least, our main goal is to find a counter-example in the case of wrong refinement. Then test cases must be as “rigid” as possible to take any incorrect behavior into account.

The following items summarize the key points of the compositional schedulability analysis framework, and in which paper they were first introduced.

- Modeling real-time actors
  - Behavioral interfaces
    - Defined in [38] as drivers, i.e., only including inputs to the actor
    - Synthetic behavior of the actor (abstract from queue and methods) including both inputs and outputs [40]
    - Deadlines as schedulability requirements [38]
  - Actors with explicit scheduling policies [38]
  - Formal semantics of a closed system of actors [this paper]
- Schedulability analysis of individual actors
  - Behavioral interface as a contract between actor and its environment [38, 40]
  - Decidability because of queue size limit [38]
  - Modeling and analysis in Uppaal (introduced in [40], a complete presentation in this paper)
- Schedulability of the composition of individually schedulable actors
  - Compatibility defined in terms of refinement extended with deadlines and quiescence (introduced in [40], formally defined in this paper)
  - Counter-example oriented testing [40]
    - Soundness [proof in this paper]
    - Exhaustiveness [proof in this paper]
    - Rigidness [introduced and proved in this paper]
- A peer-to-peer case study with dynamically reconfigurable network [this paper]

*Paper structure* Section 2 provides the grounds for the approach by explaining timed automata. In Sect. 3, we explain how we model real-time actors and exemplify this by means of a peer-to-peer case study. A review of our compositional schedulability analysis technique is given in Sect. 4. Section  describes our approach to testing refinement for timed automata, which is then applied to test compatibility in the context of schedulability analysis. Related works are presented in Sect. 6. Section 7 concludes the paper.

## Preliminaries: timed automata

We base our techniques on timed automata [7] and thus can take advantage of the abundant tools available. As we choose Uppaal  [49], we tailor the definitions accordingly.

*Syntax* Let $\textit{Act}$ be a finite set of *actions*. Let *C* be a finite set of real-valued *clocks*. We define $\mathcal B(C)$, the set of clock constraints, as the set of boolean formulas built over elementary constraints $x \sim n$ and $x - y \sim n$ where $x,y \in C$, $n \in \mathbb N$, and $\sim \ \in \{ <, \le , = , \ge , > \}$, with boolean operators $\vee$, $\wedge$ and $\lnot$. A *timed automaton* *A* *over* $\textit{Act}$ *and* *C* is a tuple $(L,l_0,E,I)$ where *L* is a finite set of *locations*, with $l_0 \in L$ being the initial location. $E \subseteq L \times \mathcal B(C) \times \textit{Act} \times 2^C \times L$ is a finite set of *edges*. We write $l \xrightarrow {g,a,r} l'$ for an edge from location *l* to location $l'$ guarded by a clock constraint $g \in \mathcal B(C)$, labeled with the action $a \in \textit{Act}$ and resetting the subset *r* of *C*. Finally, $I : L \rightarrow \mathcal B(C)$ assigns an *invariant* to each location. Location invariants are restricted to conjunctions of constraints of the form $x < n$ or $x \le n$ for $x \in C$ and $n \in \mathbb N$.

*Semantics* A timed automaton defines an infinite labeled transition system whose states are pairs (*l*, *u*) where $l \in L$ and $u : C \rightarrow \mathbb R_+$ is a *clock assignment*. We denote by $\mathbf {0}$ the assignment mapping every clock in *C* to 0. The initial state is $s_0 = (l_0,\mathbf {0})$. There are two types of transitions: action transitions $(l,u) \mathop {\rightarrow }\limits ^{a} (l',u')$ where $a \in \textit{Act}$, if there exists $l \xrightarrow {g,a,r} l'$ such that *u* satisfies the guard *g*, $u'$ is obtained by resetting to zero all clocks in *r* and leaving the others unchanged and $u'$ satisfies the invariant of location $l'$; delay transitions $(l,u) \mathop {\rightarrow }\limits ^{d} (l,u')$ where $d \in \mathbb R_+$, if $u'$ is obtained by delaying every clock for *d* time units and for each $0 \le d' \le d$, $u'$ satisfies the invariant of location *l*.

For a sequence of labels $w = w_1w_2 \dots w_n$, we write $s_0 \xrightarrow {w} s_n$ to denote the sequence of transitions $s_0 \xrightarrow {w_1} s_1 \rightarrow \dots \xrightarrow {w_n} s_n$.

*Deterministic timed automata* We call a timed automaton *deterministic* if and only if given any two edges $l \xrightarrow {g,a,r} l'$ and $l \xrightarrow {g',a,r'} l''$, the guards *g* and $g'$ are disjoint (i.e. $g \wedge g'$ is unsatisfiable). Furthermore, there is at most one transition with an invisible action $l \xrightarrow {g,\tau ,r} l'$ from any location *l*, in which case, *g* is disjoint from guards of other transitions from *l*. Note that deterministic timed automata may still produce nondeterministic behavior in the sense that at a given state, multiple transitions may be enabled, but only if they have different actions.

*Variables* As accepted in Uppaal, we allow variables of type boolean and bounded integers for each automaton. Variables can appear in guards and updates. The semantics of timed automata changes such that each state will include the current values of the variables as well, i.e. (*l*, *u*, *v*) with *v* a variable assignment. An action transition $(l,u,v) \mathop {\rightarrow }\limits ^{a} (l',u',v')$ additionally requires *v* and $v'$ to be considered in the corresponding guard and update.

*Timed automata with inputs and outputs* In the following, we assume the set of actions $\textit{Act}$ is partitioned into two disjoints sets: a set $\textit{Act} _I$ of *input actions* *a*? and a set $\textit{Act} _O$ of *output actions* *a*!. A *non-observable internal action* $\tau$ is also assumed. A timed automaton with inputs and outputs is a timed automaton over $\textit{Act} _\tau =\textit{Act} \cup \{\tau \}$.

*Network of timed automata* A system may be described as a collection of timed automata with inputs and outputs $A_i$ ($1 \le i \le n$) communicating with each other. The behavior of the system, referred to as the product or network of these automata, is then defined as the parallel composition of $A_1 \parallel \dots \parallel A_n$. Semantically, the system can delay if all automata can delay and can perform an action if one of the automata can perform an internal action or if two automata can synchronize on complementary actions (inputs and outputs are complementary). Notice that a network of deterministic timed automata is not in general necessarily deterministic. In a network of timed automata, variables can be defined locally for one automaton, globally (shared between all automata), or as parameters to the automata.

Using terminology of Uppaal, a location can be marked *urgent* in an automaton to indicate that the automaton cannot spend any time in that location. This is equivalent to resetting a fresh clock *x* in all of its incoming edges and adding an invariant $x \le 0$ to the location. In a network of timed automata, the enabled transitions from an urgent location may be interleaved with the enabled transitions from other automata (while time is frozen). Like urgent locations, *committed* locations freeze time; furthermore, if any process is in a committed location, the next step must involve an edge from one of the committed locations.

*Timed traces* A *timed sequence* $\sigma \in (\textit{Act} _{\tau } \cup \mathbb R_+)^*$ is a sequence of timed actions in the form of $\sigma = t_1 a_1 t_2 a_2 \dots a_n t_{n+1}$ such that for all *i*, $1 \le i \le n$, $t_i \le t_{i+1}$. Given a timed sequence $\sigma$, $\pi _\textit{obs} (\sigma )$ denotes the timed sequence obtained after deleting $t_i\tau$ occurrences. The sequence $\pi _\textit{obs} (\sigma )$ is called the *observable timed sequence* associated to $\sigma$.

A *run* of a timed automaton *A* from initial state $(l_0,\mathbf {0})$ over a timed sequence $\sigma = t_1a_1t_2a_2 \dots a_nt_{n+1}$ is a sequence of transitions:

$$\begin{aligned} (l_0,\mathbf {0}) \mathop {\rightarrow }\limits ^{d_1} (l_0,u'_0) \mathop {\rightarrow }\limits ^{a_1} (l_1,u_1) \mathop {\rightarrow }\limits ^{d_2} \dots \mathop {\rightarrow }\limits ^{a_n} (l_n,u_n) \mathop {\rightarrow }\limits ^{d_{n+1}} (l_n,u'_n) \end{aligned}$$

where $d_1=t_1$ and for all *i*, $1 < i \le n+1$, $t_i=t_{i-1} + d_i$. The set $\textit{Traces} (A)$ of timed traces of *A* is the set of timed sequences $\sigma$ for which there exists a run of *A* over $\sigma$. The set $\textit{Traces} _\textit{obs} (A)$ of observable timed traces of *A* is the set $\{ \pi _\textit{obs} (\sigma ) \ |\ \sigma \in \textit{Traces} (A) \}$.

## Actors as real-time asynchronous concurrent objects

We describe in this section how to use automata theory, along the lines of our previous work [38, 40], to describe actors. Actors in our framework specify local scheduling strategies, e.g., based on fixed priorities, earliest deadline first, or a combination of such policies. Real-time actors may need certain customized scheduling strategies in order to meet their QoS requirements. Our approach can be easily adapted to any actor-based modeling platform, e.g., Rebeca [1, 63], Creol [42], which in turn may provide abstractions on programs in actor-based languages like Scala or Erlang.

### A formal model of actors

An actor must be modeled at two levels of abstraction (cf. Fig. 1). First a synthetic abstract behavior of the actor is given in one automaton called its *behavioral interface*. Second, a more detailed specification of the actor behavior is given in terms of its methods, each modeled as an automaton, plus a scheduling strategy. The behavioral interface presents the actor behavior in one place, contrasted to the detailed behavior specification which is scattered over the methods. At the end of this section, we will describe how composition of multiple actor instances comprises a closed system.

*Behavioral interface model* A behavioral interface specifies at a high level, and in the most general terms, how an actor behaves. It consists of the messages an actor may receive and send. A behavioral interface abstracts from specific method implementations, the message queue in the actor and the scheduling strategy. As explained later in this section, behavioral interfaces are key to compositional analysis of actors.

To formally define a behavioral interface, we assume a finite set $\mathcal {M}$ for method names. Since every message is handled by one corresponding method, we use the terms ‘method name’ and ‘message name’ interchangeably. We assume without loss of generality that for any given method *m*, there are unique actors *a* and *b* such that only *a* can send *m* and only *b* can receive *m*, i.e., messages communicated between actors are one-to-one and unidirectional. This assumption may seem restrictive as it disallows a method to be called by different actors. To overcome this, one may duplicate the method for each caller and give it a different name; alternatively, in our implementation in the next sub-section, we consider the sender and receiver of each message as part of the message name.

#### **Definition 1**

*(Behavioral interface)* A behavioral interface *B* providing a set of method names $M_B \subseteq \mathcal {M}$ is a deterministic timed automaton over alphabet $\textit{Act} ^B$ such that $\textit{Act} ^B$ is partitioned into two finite sets of actions:

- outputs: $$\textit{Act} _O^B = \{m!|m \in \mathcal {M}\wedge m \not \in M_B\}$$
- inputs: $$\textit{Act} _I^B = \{m(d)?|m \in M_B \wedge d \in \mathbb N\}$$

The number *d* associated to input actions represents a deadline. Intuitively, this is a requirement on the implementation of an actor saying that the actor should be able to finish method *m* before *d* time units. We restrict to natural numbers for deadlines, because using real numbers makes analysis of timed automata undecidable. Output actions are the methods called by this actor and should be handled by (other actors in) the environment. We choose to disallow $\tau$ transitions at the level of behavioral interfaces to guarantee determinism in their products (see the lemma below), although in theory $\tau$ transitions can be removed if clocks are not reset [10].

The semantics of a behavioral interface is defined simply as the timed traces on its action set. We define composition of behavioral interfaces as their synchronous product on complementary actions, where an output action *m*! synchronizes with input actions *m*(*d*)? and produces the action *m*(*d*) in the composed automaton.

#### **Lemma 1**

Given a set of actors, the product of their behavioral interfaces is deterministic.

#### *Proof*

Based on the definition of determinism (see Sect. 2) and due to absence of $\tau$ transitions, nondeterminism in the product of behavioral interfaces may arise only if there are two edges from the same location with the same action, say *m*(*d*). Since messages are assumed to be one-to-one, *m*! and *m*(*d*)? can each appear in only one behavioral interface. Therefore, nondeterminism in the product is only possible if there is a similar nondeterminism in the individual behavioral interfaces, which is by definition not the case.

Behavioral interfaces conceptually serve two purposes: (1) represent the actor to the environment, as explained above; (2) represent the environment to the actor. The latter function can be enabled by syntactically swapping the ! and ? signs in a behavioral interface. Thus one obtains an abstraction of the environments in which an actor may be used. In the following sections, this abstraction will be used for modeling and analysis of actors in Uppaal.

*Actor definition* An actor may implement a behavioral interface *B* by providing implementation for the methods in $M_B$. Additionally, it has an unbounded queue to store incoming messages and a scheduling policy.

#### **Definition 2**

*(State of Queue)*: One state of a queue defined over a set of messages *M* is a finite list of triples *m*(*d*, *c*) where $m \in M$, $d \in \mathbb N$ is deadline of *m* and *c* is a clock.

A queue uses a clock *c* to keep track of the waiting time for message *m*. This clock is reset to zero when the message is added to the queue. This message misses its deadline when $c > d$. In the sequel, we do not distinguish between a queue as a general data structure and a particular state of the queue.

**Fig. 2:**
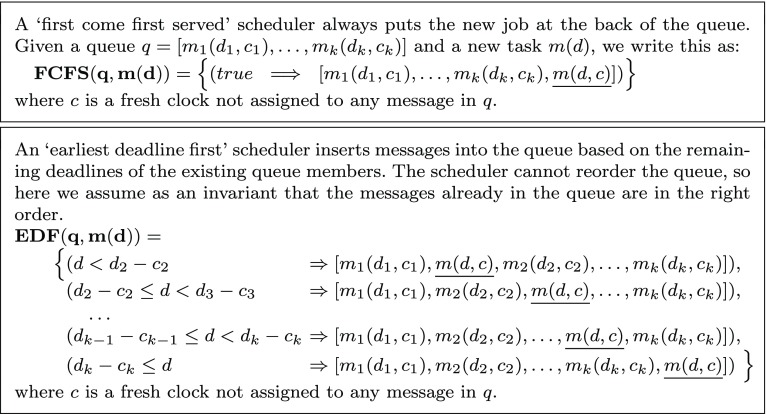
Scheduling functions acting on a queue: $q = [m_1(d_1,c_1),\dots ,m_k(d_k,c_k)]$. We write $g \Rightarrow q$ to mean that once guard *g* is satisfied the scheduler should produce the queue *q*

As part of the actor state, a queue shows the messages pending to be processed, while the first message in the queue represents the currently running method. Messages are inserted into the queue by a scheduler in the order they should be executed, based on a scheduling strategy, e.g., FCFS (First Come First Served) or EDF (Earliest Deadline First). Typically the scheduler could dynamically examine the remaining time before the deadline of each message in the queue. However, to be able to statically write down the specification of a scheduler, we define a scheduler function that returns the set of all possibilities for putting the new message in the queue depending on different clock values. Examples of such functions are given in Fig. 2.

#### **Definition 3**

*(Scheduler function)* A scheduler function acting on a set *Q* of queues defined over messages *M* is a function ‘$$ sched (q,m(d))$$’ that given $$q \in Q$$ and $$m \in M$$ with deadline *d*, returns a set of triples $$\{ (G,c,q') \}$$, where

- *G* is a guard on clocks in *q* (possibly based on *d*);
- *c* is a fresh clock not used (i.e., not assigned to any messages) in *q*; and,
- $q' \in Q$ is the queue after inserting *m*(*d*, *c*) in a particular position as implied by the guard *G*.

An overloading of the scheduler function is defined as *sched*(*q*, *m*(*d*, *c*)) such that it inserts a task into the queue using a given clock *c*. By reusing the deadline and the clock already assigned to a task in the queue, we can model inheriting the deadline. A scheduler function is $preemptive$ if it can place the new task in the first position. As discussed in [38], we only consider non-preemptive schedulers because preemption leads to undecidability. In our implementation, we will use a timed automaton to act as both the queue and the scheduler function (cf. Sect. 3.2).

#### **Definition 4**

*(Actor)* An actor *R* implementing the behavioral interface *B* is a tuple $<M_R, A_R, Q_R, S_R>$ where:

- $M_R=\{m_1,\dots ,m_n\} \subseteq \mathcal {M}$ is a set of method names such that $M_B \subseteq M_R$;
- $A_R = \{A_1,\dots ,A_n\}$ such that $A_i$ is a timed automaton implementing method $m_i$ with the alphabet $\textit{Act} _i \cup \{\tau \}$ such that $\textit{Act} _i = \{m! | m \in M_R\} \cup \{m(d)!~ |~ m \in \mathcal {M}\wedge d \in \mathbb N\}$;
- $Q_R$ is the set of all queues defined over $M_R$ and defines the set of all possible valuations of the actor’s queue; and,
- $S_R$ is a scheduling function acting on $Q_R$.

There is always ${initial}_R \in M_R$ that is responsible for initialization of the actor.

Method automata $A_i \in A_R$ only send messages while computations are abstracted into time delays. Sending a message $m \in M_R$ is called a self call. A self call with no explicit deadline inherits the (remaining) deadline of the method that triggers it (as described above using the overloaded scheduler definition); this mechanism is called delegation. Other send operations are to be given explicit deadlines. Finally note that unlike behavioral interfaces, actor automata can be nondeterministic possibly due to the $\tau$ transitions in the methods.

The semantics of an actor can be defined as a timed automaton, called *actor automaton*. Every location of the actor automaton is written as a pair (*l*, *q*) where *q* represents the contents of the queue and *l* refers to the current location of the currently executing method, i.e., the first method in the queue. The actor takes one transition if the currently running method takes a step. On the other hand, changes to the queue also cause a transition in the actor automaton. This may be receiving a new message or removing a message from the queue after it has been processed.

To concretely define semantics of an actor, one needs to characterize its environment. As mentioned earlier, the behavioral interface can act as an abstract representation of the environment. In [38], such semantics is defined and it forms the basis of individual actor analysis in Sect. 4.1. Alternatively, we define below the semantics of actors in a closed system.

#### **Definition 5**

*(Schedulable actor)* An actor is schedulable if it never reaches a state in which the queue contains a triple *m*(*d*, *c*) such that $d < c$.

In this paper, an actor is said to be *schedulable* if and only if it finishes all of the tasks within their deadlines, assuming the specific scheduling policy that is given for the actor. In principle, actors have infinite queues, but we have shown in [38] that in a schedulable system they do not put more than $\lceil d_{max} / b_{min} \rceil$ messages in their queues, where $d_{max}$ is the longest deadline for the messages and $b_{min}$ is the shortest termination time of its method automata.

#### **Lemma 2**

(Queue length) An actor with an unbounded queue is schedulable if and only if the actor is schedulable with a queue length of $\lceil d_{max} / b_{min} \rceil$.

#### *Proof*

The “if” part is trivial, so we prove the “only if” part. Assume an actor with unbounded queue has $$n = \lceil d_{max} / b_{min} \rceil + 1$$ messages in the queue. Processing them takes at least $$ n * b_{min}$$ time units and that is then longer than $$d_{max}$$. In other words, the last message finishes after more than $$d_{max}$$ time units since its creation and therefore misses its deadline.

One can calculate the best case runtime for timed automata as shown by Courcoubetis and Yannakakis [22]. This is important because finite queues make it possible to use model checking techniques for schedulability analysis (see Sect. 4).

*System composition* We define a system as a number of actors that run concurrently, each maintaining a queue of the messages it has to process. The system must be closed, i.e., any output actions by an actor must be either a self call or included in the the behavioral interface of another actor in the system.

#### **Definition 6**

*(Closed system)* A set of actors $R_1, \dots , R_n$ (as in Definition 4) with behavioral interfaces $B_1, \dots , B_n$ (as in Definition 1) comprise a closed system if for all $R_i$ we have $\textit{Act} _i \subseteq \big ( M_{R_i} \cup \bigcup _{1 \le i \le n} M_{B_i} \big )$.

**Fig. 3:**
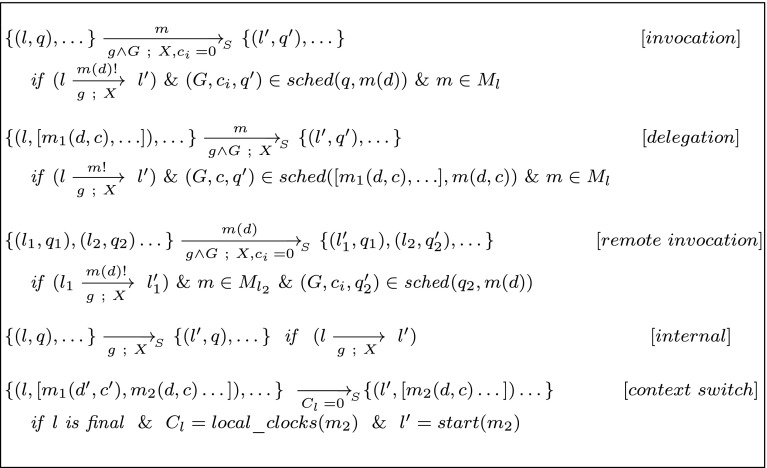
Reductions for a system

The semantics of a system is defined by a timed automaton, called the system automaton. The *system automaton* for a given system with method names $\mathcal {M}_S=\bigcup _{1 \le i \le n} M_{R_i}$ and a scheduler function *sched* is a timed automaton $S = (L_S, l_S, E_S, I_S)$ over the alphabet $\textit{Act} _S=\mathcal {M}_S$ and the clocks $C_S$:

- The set of clocks $C_S$ is the union of all sets of clocks for the method automata plus the queue clocks of each actor.
- The locations of the system are the product of each of the locations of actors together with a queue, i.e., $\{~(l_1,q_1),(l_2,q_2),\dots ,(l_n,q_n)~\}$.
- The initial location $l_S$ is:
  $$\begin{aligned} \{~( start (A_1),[m_1(d_1,c_1)]),\dots ,( start (A_n),[m_n(d_n,c_n)])~\} \end{aligned}$$
  where $$m_i$$ is $$ {initial}_{R_i}$$, $$A_i$$ is the automata corresponding to $$m_i$$ and $$ {start}(A_i)$$ is its initial location, $$d_i$$ is its deadline and $$c_i$$ is a clock.
- The edges $E_S$ are defined with the rules in Fig. 3 ($M_l$ is the set of methods provided by the actor that is in location *l*). We write $\xrightarrow [g~;~r]{m} -_{_S}$ for an edge of *S* with action *m*, guard *g* and update *r*.
- The invariant of a location is defined as the conjunction of the location invariants of all currently executing actor locations.

In Fig. 3, function *start*(*m*) returns the initial location of the automaton for method *m*. Locations in method automata with no outgoing transitions are called *final*. The first three rules in this figure take care of enqueuing sent messages. The first two are for self calls and therefore the queue of the same object is used. In case of delegation, the clock of the current task is reused and thus the deadline is inherited. Whenever the execution of the current task finishes, the context switch rule makes sure the next method in the queue is executed, if there is any. If in a location $\{(l,q),\dots \}$, *l* is final and *q* has more than one element, the location is marked urgent. This forces context switch to happen as soon as it is possible.

#### **Definition 7**

*(Schedulable system)* A closed system is schedulable if non of its constituent actors reaches a state in which the queue contains a triple *m*(*d*, *c*) such that $d < c$.

**Fig. 4:**
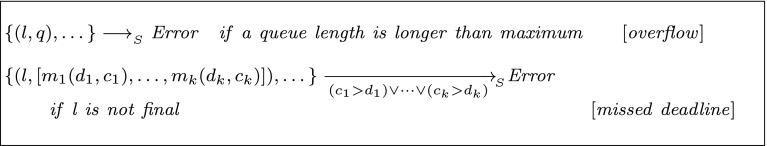
Missing a deadline leads to an error

Lemma 2 still applies in the context of a closed system. Therefore, we can put a maximum on the queue length and ensure the rules in Fig. 3 are used only if the queue bound is not exceeded. Going beyond the maximum queue length for any actor or missing a deadline by a message in a queue must also lead to an error state. The extra rules in Fig. 4 depict cases of nonschedulability by moving to an explicit error state.

In the following, we explain how to use Uppaal to implement actors and behavioral interfaces using an example.

### Modeling in Uppaal: a peer-to-peer case study

Peer-to-peer systems are a commonly used way of sharing data as well as chat-based communication. Contrasted to a client-server architecture, these systems are called peer-to-peer because all nodes can act both as a server and a client; simply said, they are all peers. We model and analyze a hybrid peer-to-peer architecture (like in Skype or BitTorrent), where a central server (called the *broker* or *tracker*) keeps track of all active nodes in the system.^1^

To start communication, a node acts as a client and asks the broker to connect it to another node. In case of Skype, the client provides the Skype ID to the broker. In a file-sharing system, a keyword is provided in order to search for some data, for example, the name of a song. The broker connects the client to a proper server, e.g., with the given Skype ID, or having the song with the given name. The two nodes then communicate directly by sending requests and replies.

Each node, upon creation, registers its ID/data with the broker. In the case of a file-sharing system, the nodes may obtain new data after every round of communication with other nodes. In this case, they need to update their information registered at the broker.

In Uppaal, we model for each actor three parts: the behavioral interface, the methods and the scheduler (which in turn includes a queue). These automata are parameterized on the identity of the actor itself (written as self), and the identifiers of the actors communicating with it (called its known actors). In this case, the known actor of a peer is a broker, and a broker has some Peers as its known actors. To have more than one instance of an actor, we instantiate the scheduler and method automata and provide different identity values (i.e., self) to different actor instances.

*Communication* In Uppaal, communication between automata is done via *channels*. We use the channels invoke and delegate for sending messages. The channel invoke has three dimensions (parameters), the message name, the sender and the receiver, e.g., invoke[connect][Peer][self]!. This way, actors instantiated from the same automata will have disjoint method names by assigning different identities to their self parameter. By setting both sender and receiver as self (in method automata), one can invoke a self call (when a deadline is to be given, as explained next). The delegate channel is used for delegation. The self call made using the delegate channel inherits the deadline of the currently running method (it is taken care of by the scheduler automaton). Since a delegation is used only for self calls, no sender is specified (it has only two parameters).

*Deadlines and parameters* We take advantage of the fact that when two edges synchronize, Uppaal performs the updates on the emitter before the receiver. Hence we can use global variables for passing information. In this case study, we use variables deadline and srv to pass deadlines and the parameter to SReq message (cf. Sect. 3.2.2), respectively. The emitter sets the desired value into the corresponding variable which is read by the receiver. The receiver, however, cannot use this value in its guard, as guards are evaluated before updates. We define these variables as *meta*, i.e., they are not kept in the state, which implies that their values must be stored properly by the receiver.

#### Behavioral interfaces

The first thing to model for an actor is its behavioral interface. Following the explanation in the previous subsection, we model a behavioral interface to represent the environment to the actor. To enable synchronization between outputs of method automata and output actions of the behavioral interface (and similarly between inputs of the scheduler and inputs of the behavioral interface), we use the ! sign for inputs and ? for outputs of the behavioral interface.

**Fig. 5:**
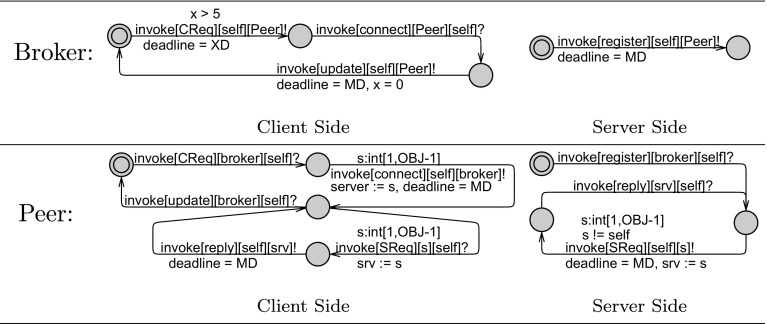
Behavioral interfaces for broker and peer as interleaving of client-side and server-side automata

Both the broker and peers have relatively independent behavior on their server side and client side. Therefore, we model these two sides using independent automata in Fig. 5, which need to be interleaved in order to produce the complete behavior of the actor. Since the messages sent by different peers to the broker are also independent, the client and server side automata of the broker are defined per peer. Figure 5 shows the messages that a broker object may use to communicate with one peer.

On the server side, the broker specifies only that a peer must register its local information once. However, on the client side, the broker expects to receive requests (CReq messages) from a peer repeatedly. For each request, the broker connects it to a server, i.e., the ID of the server is sent as a parameter of the connect message to the client; outgoing parameters are not captured in the behavioral interface. For simplicity, we assume that a request by a client is always successful, i.e., every data item searched for is available. The connections between peers is transparent to the broker. Assuming that the client peer has obtained new data after this connection, it should update its registry at the broker (because it can now provide more data on its server side). The clock x is used to ensure a delay of at least 5 time units between sending the update message and the subsequent request.

Similarly, the server side behavioral interface of a peer starts by registering its data with the broker to initialize its operation. Then it can receive requests (SReq messages) and send replies to other peers. We opt for a simple scenario, i.e., each server or client handles only one request at a time. The peer may accept an SReq message from any peer (s:int[1,OBJ-1]) excluding itself (s != self). It may only send a reply message to the same peer; this is ensured by means of the srv variable.

The behavioral interface of the peer is similar to broker on the client side, too, except that it additionally models the communication with a server after a connection has been established. The client can send any number of requests per connection, although only one at a time. Furthermore, the incoming parameter of the connect message is also captured with a select expression s:int[1,OBJ-1] in Uppaal, which means that it may receive the ID of any peer. The global variable server is used for communicating this parameter (like the deadline variable).

**Fig. 6:**
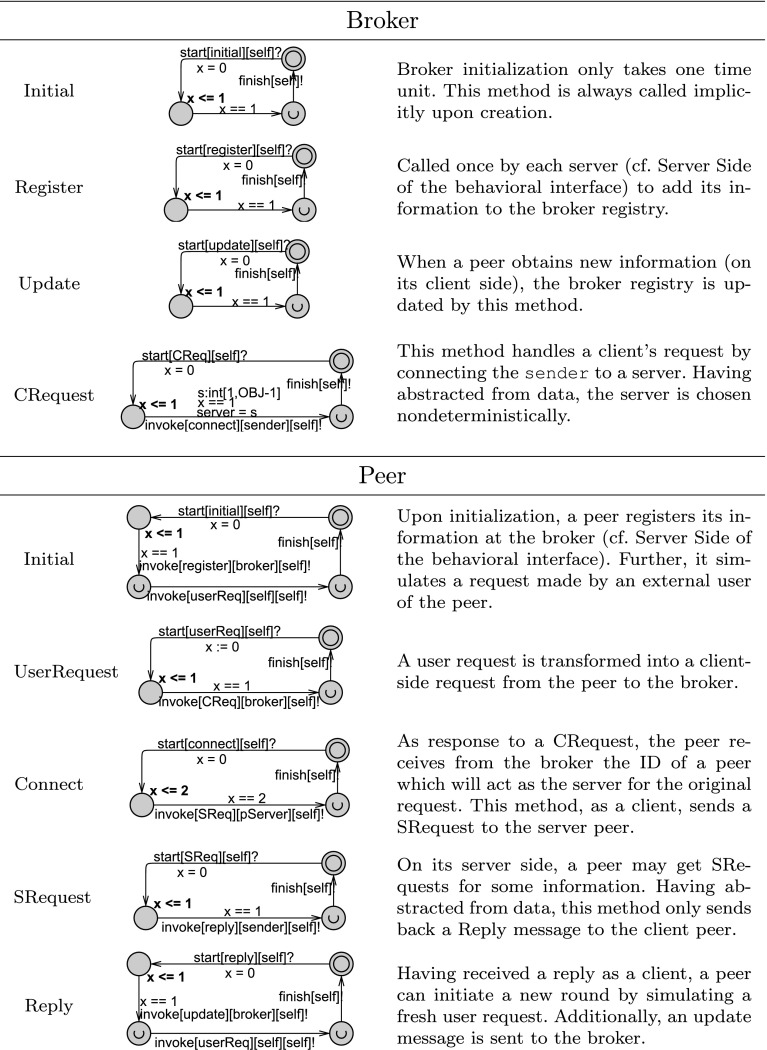
Method automata defining the broker and peer actors using the clock x to specify timing constraints

#### Broker and peer actors

Figure 6 shows the method automata of the broker and peer. In this implementation, each method is modeled as a separate automaton. A method may start its behavior when it receives a signal on the start channel from the scheduler. After accomplishing its tasks, it sends a signal on the finish channel to the scheduler, who will select the next method for execution (see next subsection).

The initial, register and update methods take one time unit to execute. These methods do not perform any computation as we abstract from the data. The CReq method nondeterministically selects a server and sends a connect message back to the sender. The variable sender is set by the scheduler to refer to the sender of a message. The ID of the selected server is sent using the server variable.

In addition to initial, a peer implements the connect and reply methods as a client, and the SReq method as a server. Furthermore, the method userReq simulates a user who initiates a search request by sending a CReq message to the broker. The userReq message is sent first by the initial method and then by the reply method in order to create a loop. Notice that this implementation of a peer sends exactly one request per connection, while the behavioral interface allows for any number of requests.

#### Modeling the scheduler

**Fig. 7:**
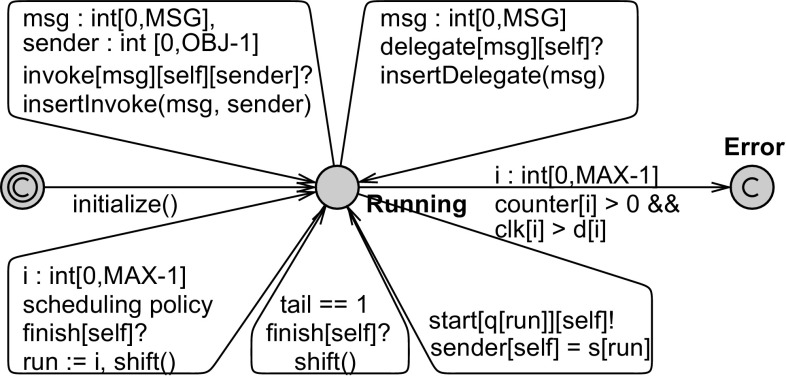
A general scheduler automaton (repeated from the main text)

A scheduler function, as described in the previous section, can be implemented as a scheduler automaton. This automaton also contains a queue. Figure 7 shows the general structure of a scheduler automaton. This general picture does not specify any specific scheduling strategy. The scheduler automata applies the scheduling strategy at dispatch time (instead of insertion time like in Definition 3), but since we only deal with non-preemptive schedulers, the resulting behavior, i.e., the order of processing messages, is the same. The reason is to enable using deadlines in the strategy. As explained earlier, the deadline value cannot be used (in the guard) on the same transition where a message is received.

*Queue* The *queue* is modeled using arrays in Uppaal and thus it can be modeled compactly, i.e., without different locations for different queue states. Tasks in the queue are modeled using the following arrays: q holds the message names, d holds their initial deadline values and clk consists of clocks that keep track of the time a task has been in the queue. The sender of every message is stored in the s array. If messages can have parameters, p arrays will be added for each parameter. We assume a maximum length of MAX for these arrays. As described in Sect. 4, we can find such a maximum for queues of schedulable objects. The array ca shows the clock assigned to each message (task), such that ‘d[ca[i]] - clk[ca[i]]’ represents the remaining deadline of q[i] at any time. counter[i] holds the number of tasks using clock clk[i]. A clock is free if its counter is zero. When delegation is used, the counter becomes greater than one.

**Fig. 8:**
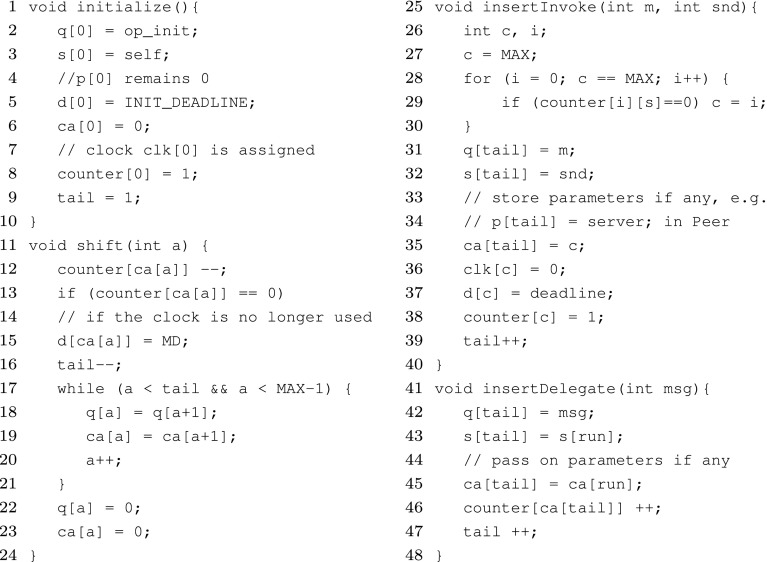
Inserting a message into the queue using invoke and delegate mechanisms

*Initializaton* The initialization of a queue takes place in the initialize function. This transition is taken before any method in any actor is started, because its start location is committed. The function shown in Fig. 8 puts the initialization method op_init in the queue and assigns a free clock to it.

*Input-enabledness* A scheduler for a class *R* should allow receiving any message in $M_R$ at any time. In Fig. 7, there is an edge (top-left in the picture) that allows receiving a message on the invoke channel (from any sender). To allow any message and sender, ‘select’ expressions are used. The expression msg : int[0,MSG] nondeterministically selects a value between 0 and MSG for msg. This is equivalent to adding a transition for each value of msg. Similarly, any sender (sender : int[0,OBJ-1]) can be selected. This message is put at the tail of the queue (q[tail] = msg), and a free clock (counter[c] == 0) is assigned to it (ca[tail] = c), and the deadline value is recorded (d[c] = deadline); this is handled in the function insertInvoke shown in Fig. 8. The synchronization between this transition and the method automata corresponds to the $invocation$ rules in Fig. 3.

A similar transition accepts messages on the delegate channel (top-right in the picture). In this case, the clock already assigned to the currently running task (parent task) is assigned to the internal task (ca[tail] = ca[run]); this is handled in the function insertDelegate shown in Fig. 8. In a delegated task, no sender is specified (it is always self). The variable run shows the index of the currently running task in the queue (which is not necessarily the first task). This handles the rule $delegation$ in Fig. 3.

*Error* The scheduler automaton moves to the Error state if a deadline is missed (clk[i] $\small {>}$ d[i]). The guard counter[i] $\small {>}$ 0 checks whether the corresponding clock is currently in use, i.e., assigned to a message in the queue. Furthermore, to make sure no queue overflow occurs, the property to check should include $tail \le MAX$.

*Scheduling strategy* When a message is added to an empty queue, the corresponding method is immediately started. When a method is finished (synchronizing on finish channel), it is taken out of the queue (by shift() given in Fig. 8). If the currently running method is the last in the queue, nothing needs to be selected (i.e., if tail == 1 we only need to shift). Otherwise, the next method to be executed should be chosen based on a specific scheduling strategy (by assigning the right value to run). For a *concrete* scheduler, the guard and update of run should be well defined. If run is always assigned 0 during context switch, the automaton serves as a First Come First Served (FCFS) scheduler. In an FCFS scheduler, the two transitions on finish channel can be combined.

A Fixed Priority Scheduler (FPS) can be implemented by associating a constant priority value to each method/task type. Suppose the array p represents the static priority of all methods, such that for a message q[i] in the queue, its priority can be obtained by p[q[i]]. We can then formulate FPS strategy using the guard: 
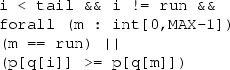


This formula selects i such that q[i] is not an empty queue cell (i  tail) or the currently finished method (run), and p[q[i]] is the highest priority. If there are multiple tasks with the same priority, the following can be added to the above guard to ensure the first task is selected: 
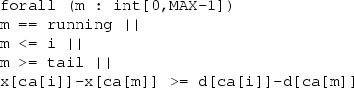


An Earliest Deadline First (EDF) scheduler always selects the task with the smallest remaining deadline. This is an example of dynamic priority scheduling because the remaining deadline of a task gets smaller as time passes. The remaining deadline of message i is given by d[i] - clk[i]. This can be encoded using a guard like: 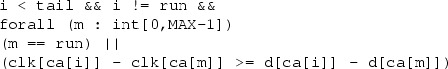


and *i* will show the task with the smallest remaining deadline. Notice that $clk[a] - clk[m] \ge d[a] - d[m]$ is equivalent to $d[m] - clk[m] \ge d[a] - clk[a]$. The rest ensures that an empty queue cell (i $\small {<}$ tail) or the currently finished method (run) is not selected.

After the next method to execute is selected, context-switch happens by starting the selected method. Having defined start as an urgent channel, the next method is immediately scheduled (if queue is not empty) by taking the bottom-right transition in Fig. 7.

## Compositional schedulability analysis

We can use the value $\lceil d_{max} / b_{min} \rceil$ for bounding the queues of schedulers as explained in the previous section. These automata have a special location Error such that a missed deadline results in an error. The system is then schedulable if the Error location is not reachable and no queue overflow occurs. In other words, schedulability analysis is reduced to reachability analysis in a tool like Uppaal and thus it is decidable. However, the intrinsic asynchrony of actors and their message buffers will lead to state space explosion for larger systems. This can be avoided by compositional analysis of the actors. To this end, we use the behavioral interface of every actor as a *contract* between the actor and its environment. Below, we describe how to check whether, firstly the actor itself, and secondly the environment in which it is used, respect this contract. We refer to the latter as compatibility check.

### Individual actor analysis

Analyzing actors in isolation is hindered by the fact that the methods of an actor can in theory be called in infinitely many ways. However, taking the behavioral interface as the contract to which the actor should adhere, it is reasonable to restrict only to the incoming method calls specified in its behavioral interface. In other words, we use the behavioral interface as a driver where the input actions correspond to the incoming messages. Incoming messages are buffered in the actor; this can be interpreted as creating a new task for handling that message. The behavioral interface doesn’t capture the internal tasks triggered by self calls. Therefore, one needs to consider both the internal tasks and the tasks triggered by the behavioral interface, which abstractly models the acceptable environments.

The semantics of an isolated actor can be defined as an *isolated actor automaton*. The states of this automaton are written as $(l_i, q_i, b_i)$ where $l_i$ shows the current location of the currently running method, $q_i$ reflects the contents of the actor queue, and $b_i$ is the current location of the behavioral interface; a full characterization is given in [38].

*Peers and broker* To analyze the broker, we need to know from how many peers it may receive requests. The automata representing the behavioral interfaces of the broker (cf. Fig. 5) need to be replicated for every peer, and these instances should be interleaved. For more efficient analysis, queue sizes smaller than $\lceil d_{max} / b_{min} \rceil$ can be tried first; and, to handle three peers it turns out that the broker can manage with a queue of size 7. For bigger systems, the model checking becomes very time consuming and intractable. To improve efficiency one can follow the guidelines in Credo methodology [29]. The analysis of each peer can be performed with one instance of its client side and server side behavioral interfaces.

**Table 1:** Schedulability analysis of peer and broker individually (top) and in a system (bottom). The number of known actors of a broker changes its behavioral interface and hence its analysis time

Single actor	# Known actors	Time
Peer	1 Broker	0:07
Broker	1 Peer	0:00
	2 Peers	0:02
	3 Peers	1:34:38
	4 Peers	$\infty$

Composed system	Time	
1 Peer and 1 broker	0:00	
2 Peers and 1 broker	4:02	
3 Peers and 1 broker	$\infty$	

Table 1 summarized schedulability analysis times of different configurations. The table on the left shows the analysis of individual actors. The high level of asynchrony introduced by increasing the number of known actors for the broker results in highly exponential increase in the analysis time. For comparison, the table on the right shows how long it would take to analyze a complete system. We can see that analysis of a complete system takes a much longer time and becomes intractable faster than individual actors. The combination of model checking and testing for compositional schedulability analysis proposed in this paper is an effective method to overcome this problem.

### Compatibility check

Once an actor is proved to be schedulable with respect to its behavioral interface, it can be used as an off-the-shelf component. A system composed of individually schedulable actors is itself schedulable if the actual use of the actors in this system is compatible with their behavioral interfaces. For each actor, the behavioral interface abstractly models its observable behavior in terms of the messages it may receive and the messages it sends.

**Fig. 9:**
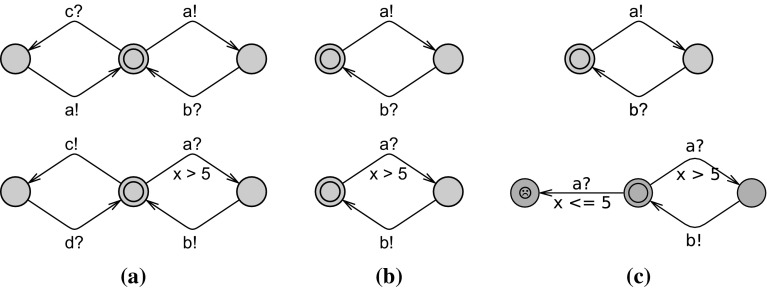
Compatibility cannot be checked at the level of behavioral interfaces

Ideally, it would be enough to check compatibility only considering the behavioral interfaces, for example, by checking deadlock freedom in the composition of the behavioral interfaces [58]. Unfortunately, this is not possible in a real-time system. We explain this using three sample pairs of behavioral interfaces shown in Fig. 9. On the one hand, behavioral interfaces cannot be used to disprove compatibility. Consider the two automata in Fig. 9a. If both automata take their left transitions, i.e., communicate by message *c*, there will be a deadlock because of the mismatching provided and required messages. However since these behavioral interfaces are abstractions of object behaviors, such a mismatch could be due to a spurious behavior not possible in the real system model. In other word, the implementations of these behavioral interfaces could happily communicate only *a* and *b* messages.

On the other hand, behavioral interfaces cannot be used to prove compatibility, either. For example, the automata in Fig. 9b can be composed with no problem, e.g., no deadlock occurs. Such a compatibility means that compatible implementations exist; but this does not guarantee compatibility of every possible implementation. An actor implementing the top interface may be too fast and send *a* outside the time constraint required by the bottom interface. In general, a behavioral interface does not reflect the precise timing of the send action by the real system model. In the work of [23], this problem is avoided by requiring the specifications to be input-enabled, like Fig. 9c where unacceptable inputs lead to an error location. This is, however, too restrictive because for example, it makes the example in Fig. 9c incompatible whereas we know already that compatible implementations exist.

The only solution to this problem is to take both the system composition and the behavioral interfaces into account. Intuitively, a running system of actors is compatible with their behavioral interfaces if its observable behavior is captured by the composition of the behavioral interfaces of the participating actors. Checking compatibility is prone to state-space explosion due to the size of the system; we avoid this by means of the testing technique described in Sect. 5. We first formally define compatibility in terms of a refinement relation.

**Fig. 10:**
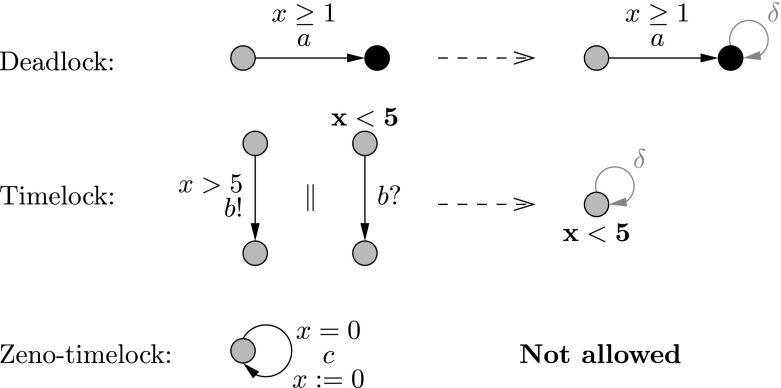
Examples of building suspension automata. We do not allow zeno-timelocks in our models

*Quiescence in refinement* In the context of timed automata, an observable behavior is either an observable action (any action except $\tau$), the passage of time (a delay) or blocking (also called quiescence, i.e., the absence of observable actions). Actions and delays are already taken into account in timed traces, which describe the possible distribution of observable actions in time. To be able to take blocking into account, we need to represent them explicitly in the specification. There are three different scenarios for blocking a real-time system:

Deadlock: No action is possible but time can go on.
Timelock: Time is stopped, no action and no delay is possible (in the left hand side of Fig. 10 the synchronization cannot happen due to the mismatching invariant and guard).
Zeno-timelock: Infinitely many actions can occur in finite time.

Deadlocks and timelocks can occur as a result of composing the behavioral interfaces, and are therefore allowed in our refinement definition; the specifications, however, should not include zeno-timelocks. Approaches like [65] can be employed to make sure zeno-timelocks do not appear in the specification. For a correct refinement, the system may deadlock (resp. timelock) only if the composition of behavioral interfaces deadlocks (resp. timelocks). Locations with a deadlock or timelock are called *quiescent*. To explicitly specify quiescence in the specification, we add a loop on each blocking location labeled by a new action $\delta$, considered to be an observable action [64] (see the right hand side of Fig. 10). The automaton obtained by adding $\delta$ actions is called a *suspension automaton*.

#### **Definition 8**

*(Suspension automaton)* Let $A = (L,l_0,E,I)$ be a timed automaton over clocks *C* and actions $\textit{Act}$. The *suspension automaton* of *A* is the timed automaton $\Delta (A) = (L,l_0,E_\Delta ,I)$ over *C* and $Act \cup \{\delta \}$ where $E_\Delta$ is defined as follows:

$$\begin{aligned} E_\Delta = E \cup \{ l \xrightarrow {\delta } l \ |\ l \text { is quiescent} \} \end{aligned}$$

The traces of the suspension automaton, called suspension traces, then represent all the observable behaviors of this automaton. The set of all suspension traces of a timed automaton *A* is the set $\textit{Traces} (\Delta (A))$ denoted by $\textit{STraces} (A)$.

In our models, *S* represents the system composition, and *B* is the synchronous product of the behavioral interfaces. Thus *B* and *S* represent two timed automata on the same set of actions, while *S* also contains $\tau$ actions. In the definition of refinement below, we consider observable suspension traces of *S* and all suspension traces of *B*.

Further we need to consider deadlines in refinement. Recall that in the behavioral interfaces, only *input* actions are assigned deadlines, whereas in the system of actors, the *output* actions in method automata have deadlines. Input actions in *B* provide the guaranteed deadlines (checked during individual actor analysis) whereas output actions in methods specify the required deadlines. In the definition of refinement below, we define that a deadline required by an action in *S* may not be smaller than the deadline guaranteed by a matching action in *B*.

#### **Definition 9**

*(Refinement)* *S* is a *refinement* of *B*, denoted by $S \sqsubseteq B$, if and only if for every observable suspension trace $\sigma = \dots , t_i, m_i(d_i), \dots$ in $\textit{STraces} _{\textit{obs}}(S)$, there exists a matching trace $\sigma ' = \dots , t_i, m_i(d_i'), \dots$ in $\textit{STraces} (B)$, such that $d_i \ge d_i'$ for every *i*.

#### **Definition 10**

*(Compatibility)* The system *S* is compatible with the behavioral interfaces if and only if $S \sqsubseteq B$ where *B* is the synchronous product of the behavioral interfaces.

The actors used in a system are proved individually schedulable with respect to their behavioral interfaces (as explained in Sect. 4.1). The following theorem states that compatibility implies schedulability of the whole system provided that individual behavioral object models are schedulable. Intuitively, this means that every message in the system will be finished within the designated deadline.

#### **Theorem 1**

(System schedulability) A system composed of a set of actors $O_1,\dots ,O_n$ is schedulable, if every actor $O_i$ is individually schedulable and the system is compatible with the behavioral interfaces of the actors.

#### *Proof*

To prove this we can assume that the system is compatible but not schedulable and all actors are individually schedulable. This means that there is a trace $\sigma = t_1,a_1, t_2,\dots ,a_k,t_{k+1}$ of the system automaton (cf. Sect. 3.1) in which one of the actors, say $O_j$, drives the system to the Error state, i.e., either the queue of $O_j$ is overflown or a task in its queue misses its deadline. We show that this requires the existence of a trace in $B_j$ that drives $O_j$ to the Error state, which contradicts the schedulability assumption.

Due to compatibility, $\sigma _{obs}$ exists in the product of behavioral interfaces. This trace can be projected onto the behavioral interface of $O_j$ alone by removing the delay-actions $t_i, a_i$ for every $a_i$ that is not in the action set of the behavioral interface of $O_j$. Call the resulting trace $\sigma _{obs}|_j$. We can compute a set of traces in ‘isolated actor automaton’ of $O_j$ (cf. Sect. 3) as $T = \{\varphi _i ~:~ \varphi _{i_{obs}} = \sigma _{obs}|_j\}$. Since the actor individually is schedulable, these traces do not lead to *Error* state, i.e., given this sequence of inputs and outputs, none of the tasks in the queue of $O_j$ misses its deadline, nor a queue overflow occurs.

On the other hand, the trace $\sigma$ corresponds to a run of the system:

$$\begin{aligned} (\{l_0^1,\dots ,l_{0}^n\},\mathbf {0}) \xrightarrow {a_1} \cdots \xrightarrow {a_{k-1}} (\{l_{k-1}^1,\dots ,l_{k-1}^n\},u_{k-1})\xrightarrow {a_k} (Error,u_k) \end{aligned}$$

where $l_i^j$ is the location of actor *j* after *i* steps. Formally, $l_i^j = (s,Q)$ where *s* is the location of its currently running task and *Q* is the current task queue. For brevity the delay transitions are not shown. We project the trace $\sigma = t_1,a_1, t_2,\dots ,a_k,t_{k+1}$ onto the actions of $O_j$, by removing the delay-actions $t_i, a_i$ such that $l_{i-1}^j = l_i^j$. We represent the resulting trace as $\sigma |_j = u_1, b_1, u_2, \dots , b_h, u_{h+1}$.

By considering the definition of system automaton, we can show that $\sigma |_j \in T$. This requires that $\sigma |_j$ drives $O_j$ to the Error state, which is in contradiction with the schedulability assumption.$\square$

Since *B* is deterministic, checking trace inclusion becomes decidable [7, 62], but due to the size of *S*, it may be susceptible to state-space explosion. To avoid this, we propose a method for *testing* trace inclusion in the next section. In particular, we want to be able to exhibit a counter-example if some incompatibility is found.

## Counter-example oriented testing: compatibility check

We will show in this section how compatibility, defined as a refinement relation based on trace inclusion, can be tested. Trace inclusion is a usual notion of correctness (or conformance) between a system and its abstract specification $\textit{B}$ in formal testing frameworks [32]. A naive approach involves taking a trace from the system model and check if it is a valid trace in the abstract specification. This is not practical because the system model is very big and is not a suitable source of generating test cases. Our idea is to generate test cases based on traces from $\textit{B}$ and use it to restrict the system behavior. As long as the system can follow this trace, the test case looks for possible violations of the refinement relation. We will formally define rigidness in this section as a characteristic of counter-example oriented testing. First, we formally define a test case.

We are given two timed automata: one is the system of actors that we call the model under test $\textit{MUT}$, and the other is the product of the behavioral interfaces of these actors. To test compatibility, we take the suspension automaton of the latter as the abstract specification. We denote this by $\textit{B}$, which is then a deterministic timed automaton over the action set $\textit{Act} _\textit{B}$. $\textit{MUT}$ is a timed automaton over the set of actions $\textit{Act} _\textit{MUT}$ = $\textit{Act} _\textit{B} \cup \{\tau \}$. A test case is a deterministic timed automaton without loops whose leaves are labeled with verdicts.

### **Definition 11**

*(Test case)* Let $\textit{B}$ be a timed automaton over $\textit{Act} _\textit{B}$. A *test case* for $\textit{B}$ is a deterministic acyclic timed automaton $\textit{TC} = (L,l_0,E,I)$ over $\textit{Act} _\textit{B} \cup \{\tau \}$, in which all leaf locations (i.e., those with no outgoing transitions) are labeled with a verdict **Pass** or **Fail**.

We refer to a set of test cases as a test set.

A verdict labeling a location allows us to evaluate an execution of the test case terminating on this location. The **Pass** verdict is reachable via only one path, which covers exactly the intended behavior we are testing for. This means that the system fulfilled the test case requirements. To find a counter-example to refinement, we need to search for locations marked **Fail**. These are the locations that are reachable with forbidden behaviors of the system (a non-specified action or an action happening outside its time constraints in the specification of $\textit{B}$, for example). If the system deviates from the behavior aimed at by the test case without violating refinement, the test may terminate prematurely resulting in an inconclusive verdict. This is similar to the ‘timed failures’ used in [59] as a semantic model for timed CSP.

Recall that proving compatibility implies schedulability of the system. However, violating refinement and thus compatibility does not per se imply the violation of schedulability. Nevertheless, considering the assume-guarantee approach, it does violate the assumptions on schedulability of individual actors specified in the behavioral interfaces. Therefore, by means of testing one can find and remove counter-examples to compatibility and, as a result, attain more confidence in schedulability of the system.

**Fig. 11:**
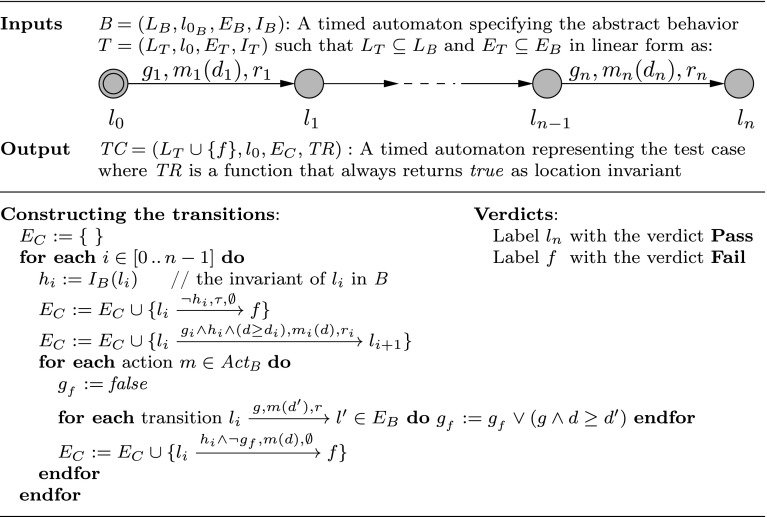
Test case generation algorithm

The fact that a test case is deterministic means that from any location *l*, for any action $a \in \textit{Act} _\textit{B}$, all transitions from *l* labeled by *a*, as well as the transition labeled by $\tau$ if any, have disjoint guards: given action $a \in \textit{Act} _\textit{B}$ and location $l \in L_\textit{B}$, then for any two guards $g_1$ and $g_2$ from the set $\{ g_i | l \xrightarrow {g_i,a,r_i} l'_i\} \cup \{ g_k | l \xrightarrow {g_k,\tau ,r_k} l'_k \}$, the formula $g_1 \wedge g_2$ is unsatisfiable.

### Generating a test case

The idea is to take a timed trace from the abstract specification $\textit{B}$ and turn it into a test case (cf. Fig. 11). Since the exact timing of actions in a timed trace make the test case too restrictive, we take instead a linear timed automaton *T*. This consists of a sequence of transitions from $\textit{B}$ representing a set of timed traces, but correspondig to exactly one untimed trace. As shown in Fig. 11, *T* contains a sequence of transitions written as $l_{i-1} \xrightarrow {g_i,m_i,r_i} l_i$, $1 \le i \le n$. Such a sequence abstractly represents a desired system behavior (the test purpose).

The sequence of transitions of *T* corresponds to the behavior we want to test so the last location must be labeled **Pass**. All other locations are completed as follows, such that any forbidden behavior makes the test fail. If a location has an invariant $h_i$ in $\textit{B}$, violating this invariant must make the test fail; thus, a transition labeled with $\tau$ and with guard $\lnot h_i$ leading to **Fail** is added. Furthermore, no other transition may be taken if the invariant is violated; this is ensured by conjunction of guards of all other transitions with $h_i$. Additionally, every behavior which is not allowed in $\textit{B}$ is forbidden, so for every action, a transition labeled by this action and whose guard is the complement of all the existing guards for this action leads to a **Fail** location; this guard is computed in $g_{_f}$. Any trace leading to **Fail** is an example of behavior not allowed in the abstract behavior specification.

**Fig. 12:**
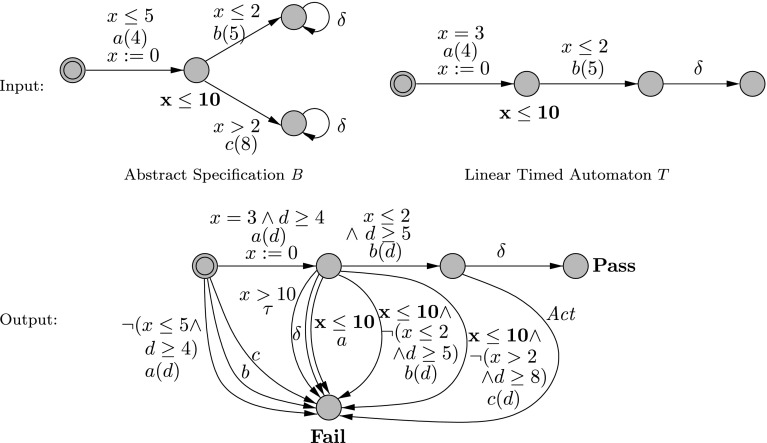
In this example, the input $\textit{B}$ is a suspension automaton. Actions with no deadlines in the output test case imply unrestricted deadline values. Label *Act* means all actions are possible

#### *Example 1*

In Fig. 12, $\textit{B}$ shows the suspension automaton of an abstract specification, *T* is a selected sequence of transitions from $\textit{B}$ and finally $\textit{TC}$ is the test case generated from *T* by the algorithm. The location invariant $\mathbf {x \le 10}$ is kept in bold face in the test case only to show its effect on guards. The transition labeled by $\textit{Act}$ stands for three transitions labeled by *a*, *b* and *c* with guard $\textit{true}$.

The theorem below states that the timed automaton we obtain by this construction is a test case in the sense of Definition 11. The proof is straightforward by following the steps in the algorithm.

#### **Theorem 2**

Let $\textit{B}$ be a timed automaton over $\textit{Act} _\textit{B}$. For any linear timed automaton from $\textit{B}$, the automaton generated using the algorithm in Fig. 11 is a test case.

*Remark.* The starting point for test case generation is a sequence of transitions in the specification. Given a desired reachability property $\varphi$, we can generate such a sequence of transitions automatically. We start by model-checking $\varphi$ on the suspension automaton of the specification $\textit{B}$. The diagnostic trace produced by the model-checking tool gives the sequence of moves that have to be made by this automaton and the required clock constraints needed to reach the targeted location. This method is in parts similar to [32]. Instead of checking for a reachability property, one can also use the simulation feature of a model-checker to generate specific hand-made traces. Another interesting property is a deadlock or timelock in the abstract specification $\textit{B}$. Although a correct refinement is theoretically allowed to deadlock or timelock in such cases, too, such situations are in practice undesired. Therefore, such traces could also contribute to good test cases for checking system correctness. Note that a timelock in our models does not violate schedulability because when time stops no deadline is missed, but such a scenario is in fact an unrealistic situation.

### Properties of the generated test cases

A test case must drive the execution of the system such that actions happen in the specified order. Usually, this happens by feeding inputs to the system and observing the outputs. In our case, we deal with a closed system which has no inputs to be controlled. Instead, since we deal with a system *model*, we drive the system execution by making it synchronize with the test case. Formally, the execution of a test case on the system is defined as the parallel composition of the automata of the test case and the system, synchronizing on the same actions. We denote the product automaton by $\textit{TC} \parallel \textit{MUT}$.

The model under test *passes* the test, denoted by $\textit{MUT} \mathsf {\ passes\ }\textit{TC}$, if and only if the **Fail** location is not reachable in the product $\textit{TC} \parallel \textit{MUT}$. A test set $\mathcal{T}$ being a set of test cases, the model under test passes $\mathcal{T}$, denoted by $\textit{MUT} \mathsf {\ passes\ }\mathcal{T}$, if and only if for all test cases $\textit{TC}$ in $\mathcal{T}$, $\textit{MUT} \mathsf {\ passes\ }\textit{TC}$.

#### Soundness

The soundness requirement for a test set states that it must not reject a correct refinement. In other words, any counter-example reported by a test case (a trace leading to the **Fail** verdict) should indeed violate the refinement. A test case is formally defined to be *sound* (or unbiased) for the refinement relation $\sqsubseteq$ if and only if

$$\begin{aligned} \textit{MUT} \sqsubseteq \textit{B} \implies \textit{MUT} \mathsf {\ passes\ }\textit{TC} \end{aligned}$$

A test set $\mathcal{T}$ is sound if and only if all test cases in $\mathcal{T}$ are sound.

##### **Theorem 3**

(Soundness) Let $\textit{B}$ be a deterministic timed automaton and *T* be a linear timed automaton built from a sequence of transitions in $\textit{B}$. The test case *TC* generated from *T* and $\textit{B}$ by the algorithm in Fig. 11 is *sound* for $\sqsubseteq$.

##### *Proof*

We assume that $\textit{MUT}$ does not pass *TC*, i.e., there is a trace in $TC \parallel \textit{MUT}$ that leads to the fail location $l_f = (l,f)$. This trace can be decomposed into its $\textit{MUT}$ and *TC* components. Every location in *TC*, except for *f*, can be mapped to a location in $\textit{B}$; since $\textit{B}$ is deterministic, this mapping is unique. The diagram below, shows the decomposition of this trace and the mapping to $\textit{B}$.We show that the last step in the trace does not exist in $\textit{B}$. This step is due to a transition to the fail location *f* of *TC* which may be due to one of the following cases, based on the test case generation algorithm in Fig. 11.

- It might be a $\tau$ transition which implies that the delay $d_{i}$ is not permitted by the invariant of $l_i^{^S}$ in $\textit{B}$; or,
- It might be that the action $a_{i}$ that happens at $u_{i}^{^T} + d_{i}$ leads *TC* to *f*, i.e., $u_{i}^{^T}$ satisfies the guard $\lnot g_f$ where $g_f$ is the disjunction of all guards that allow $a_{i}$. As a result, action $a_{i}$ is not allowed at this time in $\textit{B}$.

The trace shown above does not exist in the suspension traces of $\textit{B}$, while it obviously does exist in the observable suspension traces of $\textit{MUT}$. Therefore, $\textit{MUT}$ is not a refinement of $\textit{B}$.$\square$

#### Exhaustiveness

Soundness is not sufficient to ensure the relevance of test cases. A test case with no **Fail** location is sound but cannot reject any system. We also need to be sure that if the system is a wrong refinement, there exists a test case able to reject it. An exhaustive test set rejects any wrong refinement in the system. In other words, any system that passes the test set is a correct refinement of the specification. A test set is formally defined to be *exhaustive* for the refinement relation $\sqsubseteq$ if and only if

$$\begin{aligned} \textit{MUT} \mathsf {\ passes\ }\mathcal{T} \implies \textit{MUT} \sqsubseteq \textit{B} \end{aligned}$$

##### **Theorem 4**

(Exhaustiveness) The set of all test cases for $\textit{B}$ that can be generated by the algorithm in Fig. 11 is *exhaustive* for $\sqsubseteq$.

##### *Proof*

We must prove that if the system is not a refinement, there exists a test case that makes the system fail. In other words, assuming that there exists an observable suspension timed trace of $\textit{MUT}$ not belonging to suspension timed traces of $\textit{B}$, we must show that there exists a test case $\textit{TC}$ such that the **Fail** location is reachable in the product $\textit{TC} \parallel \textit{MUT}$.

Without loss of generality, we consider $\sigma =t_1a_1\dots t_ka_k \in \textit{STraces} _\textit{obs} (\textit{MUT})$. The corresponding observable run in $\textit{MUT}$ is the following:

$$\begin{aligned} (l'_0,\mathbf {0}) \xrightarrow {d_1} (l'_0,u'_0) \xrightarrow {a_1} (l'_1,u_1) \rightarrow \dots \xrightarrow {d_k} (l'_{k-1},u'_{k-1}) \xrightarrow {a_k} (l'_k,u_k) \end{aligned}$$

If $\sigma$ is not a trace of $\textit{B}$, then two cases are possible depending on the first behavior diverging from the trace of $\textit{B}$:

1. There exists *i*, $0 \le i \le k-1$ such that $t_1a_1\dots t_ia_i \in \textit{STraces} (\textit{B})$ and $t_1a_1\dots t_ia_it_{i+1} \notin \textit{STraces} (\textit{B})$, i.e., a delay of $d_{i+1}=t_{i+1}-t_i$ is not possible in $\textit{B}$ at state $(l_i,u_i)$. It means that location $l_i$ has an invariant *h* that is violated by $u_i+d_{i+1}$. Let *T* be a sequence of $i+1$ transitions from $\textit{B}$ such that $t_1a_1\dots t_ia_i \in \textit{STraces} (T)$. Let $\textit{TC}$ be the test case generated from *T* by the algorithm. Since location $l_i$ has an invariant *h*, there is a transition in $\textit{TC}$ from $l_i$ to location *f* whose guard is $\lnot h$ and labeled with $\tau$. As $u_i+d_{i+1}$ does not satisfy *h*, location *f* is reachable in the product $\textit{TC} \parallel \textit{MUT}$ with the trace $t_1a_1\dots t_ia_it_{i+1}\tau$ corresponding to the run
  $$\begin{aligned} ((l_0,l'_0),\mathbf {0}) \xrightarrow {d_1} ((l_0,l'_0),u'_0) \xrightarrow {a_1} \dots \xrightarrow {d_{i+1}} ((l_i,l'_i),u'_i) \xrightarrow {\tau } ((f,l'_{i+1}),u_{i+1}) \end{aligned}$$
2. There exists *i*, $0 \le i \le k-1$ such that $t_1a_1\dots t_ia_it_{i+1} \in \textit{STraces} (\textit{B})$ and $t_1a_1\dots t_ia_it_{i+1}a_{i+1} \notin \textit{STraces} (\textit{B})$, i.e., the action $a_{i+1}$ is not allowed in $\textit{B}$ at time $t_{i+1}$. It means that there is no transition in $\textit{B}$ from location $l_i$ labeled with $a_{i+1}$ whose guard is satisfied by $u_i+d_{i+1}$. Let *T* be a sequence of $i+1$ transitions from $\textit{B}$ such that $t_1a_1\dots t_ia_it_{i+1} \in \textit{STraces} (T)$. Let $\textit{TC}$ be the test case generated from *T* by the algorithm. By construction of $\textit{TC}$, there is a transition from location $l_i$ to location *f* labeled with $a_{i+1}$ whose guard is the complement of all other guards of transitions from $l_i$ labeled with $a_{i+1}$, let us call it *g*. Since $u_i+d_{i+1}$ does not satisfy any of these guards, it satisfies *g*. Then location *f* is reachable in the product $\textit{TC} \parallel \textit{MUT}$ with the trace $t_1a_1\dots t_ia_it_{i+1}a_{i+1}$ corresponding to the run
  $$\begin{aligned} ((l_0,l'_0),\mathbf {0}) \xrightarrow {d_1} ((l_0,l'_0),u'_0) \xrightarrow {a_1} \dots \xrightarrow {d_{i+1}} ((l_i,l'_i),u'_i) \xrightarrow {a_{i+1}} ((f,l'_{i+1}),u_{i+1}) \end{aligned}$$

Therefore, the set of all test cases generated by the algorithm is exhaustive for $\sqsubseteq$.

A sound and exhaustive test set is called *complete*. Completeness is in general impossible to reach, since it usually needs an infinite test set. Thus we know that we cannot in practice find all counter examples to refinement. However, we still want to ensure a certain quality to test cases. For instance, we want to avoid useless sound test cases where all paths lead to **Pass**. Below, we introduce a more practical property for test cases.

#### Rigidness

We are interested in test cases that reject models which behave in a wrong way *along the test case*: the test case should not say **Pass** if it is possible to detect something wrong during the test case execution. We show that any test case generated by our algorithm can detect every wrong behavior occurring along it. We can actually show that we can provide a counter-example for any incorrect refinement occurring along the sequence of transitions the test case is built from. Given a trace $\sigma \in \textit{STraces} (\textit{B})$, an incorrect refinement is formally characterized as an action or delay $e \in \textit{Act} \cup \{\delta \} \cup {\mathbb R}_+$ that is allowed after $\sigma$ in $\textit{MUT}$ but not in $\textit{B}$, i.e., . A test case $\textit{TC}$ is rigid for the refinement relation $\sqsubseteq$ if and only if it rejects any incorrect refinement along the traces of the test case:

$$\begin{aligned} \sigma \in \textit{Traces} (\textit{TC}) \wedge l_0 \xrightarrow {\sigma } l_i, 1 \le i < n \Rightarrow \sigma .e \in \textit{Traces} (\textit{TC}) \wedge l_0 \xrightarrow {\sigma .e} \mathbf{Fail} \end{aligned}$$

Intuitively, if $\sigma$ ends in a non-leaf location in $\textit{TC}$, the test case *TC* will observe any one-step divergence after $\sigma$. This notion is close to the notions of non-laxness in the untimed setting [41] and of strictness in the timed setting [46] but it is stronger. These notions state that if the system behaves in a non-conforming way during the execution of the test case, it must be rejected. Also in our framework, every detected divergence leads to the rejection of the system, but we can add that every divergence is actually detected. This result directly follows from the construction of the test case.

##### **Theorem 5**

(Rigidness) Let $\textit{B}$ be a deterministic timed automaton and *T* be a linear timed automaton built from a sequence of transitions in $\textit{B}$. The test case for $\textit{B}$ generated from *T* by the algorithm in Fig. 11 is *rigid* for $\sqsubseteq$.

##### *Proof*

We show that for every trace $\sigma$ of the test case $\textit{TC}$ ending in a non-leaf location, $\sigma .e$ is a trace of $\textit{TC}$ leading to **Fail** if $\sigma .e$ is a trace of $\textit{MUT}$ and not of $\textit{B}$.

If $e \in \textit{Act} \cup \{ \delta \}$, let $\sigma = t_1a_1 \dots t_k \in \textit{Traces} (\textit{TC})$ corresponding to the run

$$\begin{aligned} (l_0,\mathbf {0}) \mathop {\rightarrow }\limits ^{d_1} (l_0,u'_0) \mathop {\rightarrow }\limits ^{a_1} (l_1,u_1) \rightarrow \dots \mathop {\rightarrow }\limits ^{d_k} (l_{k-1},u'_{k-1}) \end{aligned}$$

where $k-1 \ne n$. Since $\sigma .e$ is not a trace of $\textit{B}$, it means that *e* is not allowed in $\textit{B}$ at location $l_{k-1}$ after a delay of $d_k$. Then, by construction of the test case $\textit{TC}$, there is a transition from $l_{k-1}$ to the **Fail** location labeled with action *e* and whose guard satisfies $u'_{k-1}$. Then $\sigma .e$ is a trace of $\textit{TC}$ and $l_0 \xrightarrow {\sigma .e} \mathbf{Fail}$.

If $e \in \mathbb R_+$, let $\sigma = t_1a_1 \dots a_k \in \textit{Traces} (\textit{TC})$ corresponding to the run

$$\begin{aligned} (l_0,\mathbf {0}) \mathop {\rightarrow }\limits ^{d_1} (l_0,u'_0) \mathop {\rightarrow }\limits ^{a_1} (l_1,u_1) \rightarrow \dots \mathop {\rightarrow }\limits ^{a_k} (l_k,u_k) \end{aligned}$$

where $k \ne n$. Since $\sigma .e$ is not a trace of $\textit{B}$, it means that a delay of *e* is not allowed in $\textit{B}$ at location $l_k$, due to an invariant *h* at this location in $\textit{B}$. Then, by construction of the test case $\textit{TC}$, there is a transition from $l_k$ to the **Fail** location labeled with $\tau$ and whose guard $\lnot h$ satisfies $u_k$. Then $\sigma .e$ is a trace of $\textit{TC}$ and $l_0 \xrightarrow {\sigma .e} \mathbf{Fail}$.$\square$

**Fig. 13:**
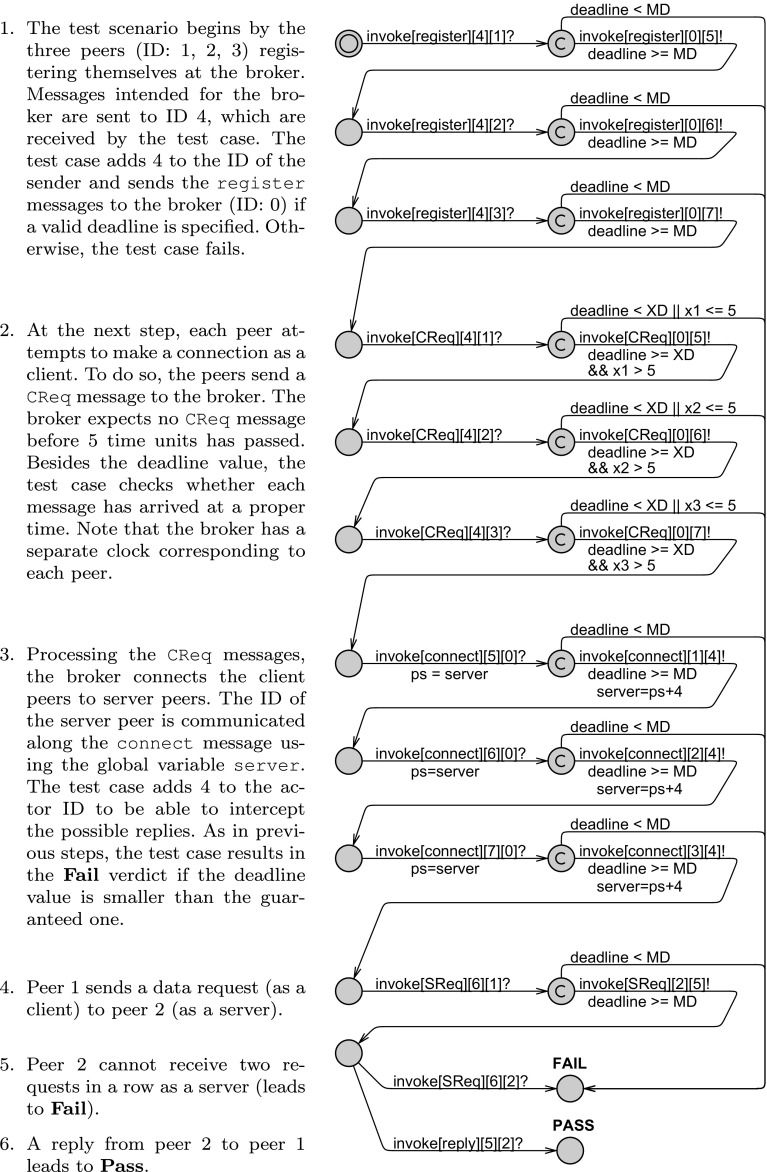
In this test case, the broker actor is assigned 0 while the peer actors are assigned 1, 2 and 3. In order to intercept the messages between actors, the test automaton represents each actor by adding 4 to its ID (when binding the known actors). For the sake of simplicity, this test case does not include all possible violations of refinement that lead to the **Fail** verdict

### Executing test cases in Uppaal

Recall that we are testing the inclusion of the observable traces of a system *S* in the traces of a specification *B*. Test cases generated from *B* are used to restrict the system behavior and at the same time detect any violations of refinement along the test case. When submitting a test case, we require that any communication between two actors should synchronize with the test case, as well. Practically, this means that the sender actor (in one of its methods), the receiver actor (in its scheduler) and the test case should synchronize. Since we do not want to change the specification of the model under test, we solve the problem of three-way synchronization by splitting every action in the test case into two steps. At the first step, the sender actor synchronizes with the test case, and *immediately* afterwards, the test case synchronizes with the receiver actor. The urgency between these two steps is modeled by using a ‘committed’ location in the test case between these two steps. For the test case to be able to intercept the messages, we bind all known actors to refer to the test case (see Fig. 13).

Although a test case is deterministic and its synchronization with the system resolves part of the non-determinism, the final model is not yet completely deterministic. The actor implementations may also contain some internal choices that are not controlled by the test case. In principle, this would mean that a test case needs to be repeated several times and a coverage of different nondeterministic choices requires extra control over the system behavior. To avoid this problem, we take advantage of the model checking capability of Uppaal as explained in the sequel.

What is important in the execution of a test case is the final verdict. Reaching a **Pass** or **Fail** verdict can in fact be formulated as a reachability property in Uppaal. This way each test case needs to be submitted once. The Uppaal model checker can provide a diagnostic trace whenever the searched verdict is reachable. A trace leading to the **Fail** verdict shows exactly how and when the system is not compatible.

Using the model checker in this scenario is plausible because the system behavior is controlled by the test case, while model-checking the whole system may not be tractable. We thus avoid state space explosion by restricting verification to the part of system behavior that follows the main line of the test case.

### Testing compatibility for the peer-to-peer system

In this section, we give a sample test case generated for a system consisting of 3 peers and a broker. We have already demonstrated in Table 1 that model checking the whole system runs out of system resources and is not feasible for 3 or more peers. The test case in Fig. 13 is generated from the composition of the behavioral interfaces of peer and broker (cf. Fig. 5) considering three peers. Execution of this test case takes less than a second.

As mentioned earlier, the server side behavioral interface of a peer allows only one request at a time. This means that two SReq messages may not be sent to the same peer before it has replied to the first one. The scenario captured in this test case is designed to check this property for peer number 2 (in Step 5).

This test case starts with registering the servers at the broker followed by requests from clients to the broker. Then the broker replies to these requests by sending via the server variable. Since server is defined as a meta variable, the test case uses a temporary variable ps in order to pass on this value to the clients. If a behavioral interface specification requires special conditions on the values of this parameter, the test case would also check these conditions. Finally, if two SReq messages are sent to peer number 2, the test fails, i.e., there is a counter-example to compatibility. The test passes if server 2 replies to the first request.

Given the nondeterministic selection of a server in the CReq method (cf. Fig. 6), the test case in Fig. 13 will fail. The reason is that the broker may assign the same server to multiple clients which may independently send a request to this server. One simple solution to this incompatibility would be using a round-robin assignment of the servers by the broker to the incoming requests. With this strategy, the test case in Fig. 13 does not lead to the **Fail** verdict anymore.

## Related work

There has been lots of work on scheduling in real-time systems [13]. The main aspect of our work is that we address schedulability at a *modeling* level as in [6, 25, 27, 56], whereas [21, 45] are applied to *programming* languages. This results in a major methodological difference. In the latter case, analysis is performed only after the software has been developed: a given application is augmented with real-time requirements (like deadlines) and automata are derived from code. This approach can be useful specifically for legacy software. In contrast, we use automata for platform-independent modeling of actors and their behavioral interfaces at the design stage. One can thus boost the application performance by fine-tuning the scheduling in the early steps of development and at a much lower cost. With this in mind, we compare our schedulability and testing methods with some most relevant works, focusing on its different aspects.

*Task specifications* Schedulability analysis results depend on the assumptions on task generation. Many approaches are based on simple task generation patterns like periodic tasks and rate-monotonic analysis [27, 61]. Although useful in many cases, such techniques are too coarse in many distributed systems and produce pessimistic outcomes. In our work, behavioral interfaces specify how tasks may be generated in an actor. Being based on Task automata [25], we can describe non-uniformly recurring tasks, inducing much more accurate analysis. We extend also the decidability results by Fersman et al. [25] and show that our analysis (based on non-preemptive scheduling) can be reduced to checking for reachability in timed automata, and is therefore decidable [31]. Although  [21, 45] also use automata, they do not discuss decidability.

The main difference between our work and task automata is that in our framework tasks are specified as timed automata. Therefore tasks can trigger other (internal) tasks during execution, which may inherit the (remaining) deadline of the task generating them (called delegation). In task automata, a task is completely abstracted away into an execution time, and generation of all tasks is captured in the task automaton. In our approach, internal tasks cannot be captured in the behavioral interfaces, because their arrival depends on the scheduling of the parent tasks, which in turn depends on the selected scheduling strategy. Our approach is therefore strictly more expressive than task automata.

*Compositionality* Schedulability has usually been analyzed for a whole system running on a single processor, whether at modeling [6, 25] or programming level [21, 45]. We address distributed systems where each actor has a dedicated processor and scheduling policy. In our approach, behavioral interfaces are key to compositionality. They model the most general message arrival pattern for actors. They can be viewed as a contract as in ‘design by contract’ [53] or as a most general assumption in modular model checking  [48] (based on assume-guarantee reasoning); schedulability is guaranteed if the actual use of the actor (i.e., at the method definition level) satisfies this assumption in the behavioral interface.

The approach in [56] is modular in the sense that the untimed specification of the actors, and the timing constraints (specified separately) can be reused. However, they still analyze a complete system, rather than individual actors. Furthermore, a deadline in their framework includes only the time until an event is received. Hence, their approach cannot address complications like delegation of a task to subtasks. Another related work is TAXYS [21], where an abstract model of the environment is used for schedulability analysis. However, it is used to analyze a complete program and is not used compositionally.

*Interface design* Our behavioral interfaces are similar to Timed Interface Automata [24], but the notion of compatibility is different. Alfaro et al. [24] take an optimistic approach in which two interfaces are compatible if there is a possible way for them to work properly. This leads to a simpler theory but to implement these interfaces, one needs to adhere to these possibilities to end up with a working system. David et al. [23] suggest to make specifications input-enabled by adding an Error state and directing every undesired behavior to that state. They define two specifications to be compatible if their composition does not reach the Error state. This is unfortunately too restrictive for high-level specifications; abstract behavioral interfaces easily fall into spurious incompatibilities whereas their implementations may still work together. Our approach bridges the gap between these two methods by considering the actual implementation of actors. We check whether the implementations at hand, when composed, indeed follow the behavior that makes their interfaces compatible (w.r.t. the optimistic approach of [24]). Finally, timed actor interfaces in [28] are defined to accept *early* actions. This is an orthogonal feature and can be combined with our notion of behavioral interfaces if desired.

Analyzing the composition of the concurrent objects is subject to state space explosion because of their asynchronous nature and all their queues. We discussed in [36] the necessary conditions for compositional model checking of refinement. However it is not sufficient to prove refinement in all cases. We proposed in this paper a sound and complete testing technique for compatibility, based on finding counter-examples to refinement, as positioned below.

*Testing real-time systems* In real-time systems, conformance (or refinement) is tested in terms of allowed actions as well as of right timings. Different conformance relations have been investigated: timed bisimulation [14], may and must preorders [55], timed trace inclusion [15, 32, 44], and timed extension of Tretmans’ conformance relation **ioco** [47, 60, 64]. The main difference here is that our notion of refinement takes deadlines on actions into account. Similarly, timelocks (and sometimes also deadlocks) are generally considered errors in a specification. When testing, the specification and the implementation are usually assumed to be non-blocking, meaning that they will never block time in any environment. However, since our specification is obtained by synchronous product of behavioral interfaces, such cases can happen, it makes sense to allow the same “errors” in the system as in its specification: Our conformance relation then takes into account the presence of deadlocks and timelocks, and allows them in the system whenever they exist in the specification.

A naive approach to testing refinement is to take a trace from the concrete system and check whether it exists also in the abstract specification. This approach is impractical because the concrete system is too complicated. Therefore, as is usual in the literature, e.g., in ioco testing, we generate test cases from the abstract specification. A main difference is that these techniques are for testing an open system, i.e., by feeding inputs and observing the outputs. However, we deal with a closed system, i.e., we have no inputs and outputs. In this respect, we take advantage of the fact that the system under test is also a model in our case (and not an implementation for example in Java or C), so we can use a tool like Uppaal, and drive the system execution by synchronizing on the actions of the test case. This greatly restricts the system behavior, although still nondeterministic. The restricted system behavior is now amenable to model checking in order to address the remaining nondeterminism during test submission.

A similar approach of using testing techniques to avoid state space explosion in the analysis of real-time system models has been followed by Clarke and Lee [20], in the setting of a real-time process algebraic formalism called ACSR (the Algebra of Communicating Shared Resources). They focus on testing timing constraints of real-time systems, deriving time-efficient test cases from a graphical representation of those constraints and defining time domains coverage criteria.

The soundness of our testing method depends on the determinism of the behavioral interfaces. The problem of determinizability of arbitrary timed automata is undecidable [26, 66], so in this paper we require the behavioral interfaces to be given as deterministic automata. To relax this requirement, one can consider the class of determinizable timed automata as in  [44], or use digital test cases [47], where time is discrete to answer the implementability of test cases. Alternatively, automated over-approximation techniques as in [12] can be employed.

## Conclusions

The main contribution of this work is the integration of the abstract formalism of timed automata into a high-level object based modeling paradigm (along the same lines as typestate-oriented programming [5]). On the one hand, the abstraction level of automata theory enables us to provide powerful analysis techniques and specifically less pessimistic schedulability analysis compared to traditional approaches. On the other hand, we augment the successful actor-based approach to object-orientation (as in Scala and Erlang) with application-level scheduling, as also motivated in resource-aware programming techniques.

We presented a complete framework for compositional schedulability analysis of distributed systems. Schedulability of each actor is analyzed individually with respect to its behavioral interface. This is made feasible by putting a finite bound on the task queue such that the schedulability results hold for any queue length. We can then test a system of communicating objects to make sure objects are used as expected. This compatibility further implies the schedulability of the whole system. In this paper, we specifically gave a detailed account of a novel counter-example oriented technique for testing refinement as the basis for compatibility check. To this end, we gave an algorithm to generate sound, complete and rigid test cases. Overall, we envisage such maximal use of verification combined with testing as a promising approach to deal with state-space explosion problem.

As future work, we are planning to integrate this high-level analysis framework into our implementation of application-level scheduling on top of Java [57]. The integrated tool suite will span a complete software development cycle, and will be a basis for developing safety critical real-time distributed and embedded systems. The main advantage of such a tool suite is that the designer/programmer will gain direct control on scheduling in the whole development cycle, which is in turn the key to efficiency in the software running on future multi-core and cloud infrastructures. Nonetheless, it will in practice be based on a best-effort basis as true runtime guarantees depend on the exact operating system and the hardware.

A specific and interesting case where we can apply our approach is in modeling TinyOS [18, 35]. TinyOS is an actor-based open-source runtime environment designed for sensor network and has a large user base of over 500 research groups and companies [18]. The event-driven execution model of TinyOS enables fine-grained power management yet allows the scheduling flexibility made necessary by the unpredictable nature of wireless communication and physical world interfaces. Modeling a TinyOS instance as an actor, we can define its own scheduling policy and hence the designer is able to introduce and analyze different policies in scheduling.

## Footnotes

1 These Uppaal models are available at http://www.cwi.nl/~jaghouri/P2P/.
